# Maternal major depression disorder misclassification errors: Remedies for valid individual‐ and population‐level inference

**DOI:** 10.1002/brb3.2614

**Published:** 2022-05-19

**Authors:** Arthur H. Owora

**Affiliations:** ^1^ Department of Epidemiology and Biostatistics, School of Public Health Indiana University Bloomington Indiana

**Keywords:** inference, maternal MDD, MDD case‐finding tools, misclassification error

## Abstract

Individual and population level inference about risk and burden of MDD, particularly maternal MDD, is often made using case‐finding tools that are imperfect and prone to misclassification error (i.e. false positives and negatives). These errors or biases are rarely accounted for and lead to inappropriate clinical decisions, inefficient allocation of scarce resources, and poor planning of maternal MDD prevention and treatment interventions. The argument that the use of existing maternal MDD case‐finding instruments results in misclassification errors is not new; in fact, it has been argued for decades, but by and large its implications and particularly how to correct for these errors for valid inference is unexplored. Correction of the estimates of maternal MDD prevalence, case‐finding tool sensitivity and specificity is possible and should be done to inform valid individual and population‐level inferences.

## 1 MAJOR DEPRESSION DISORDER DEFINITION

Major depression disorder (MDD, also known as clinical depression) is a mental health condition characterized by a depressed mood and/or anhedonia (loss of interest or pleasure), and at least five of seven other symptoms that reflect a change in normal functioning or impaired functioning such as feelings of worthlessness or guilt, insomnia or hypersomnia, irritability, low energy levels, changes in appetite and weight, reduced concentration and suicidal thoughts almost all day, every day for at least a 2‐week period (American Psychiatric Association, 2000). This definition of MDD is symptom‐based and could be viewed as an abstract concept; making it impossible to diagnose MDD objectively. The diagnosis of MDD is further complicated by its unclear etiology and pathophysiology (Hasler, 2010).

## 2 ETIOLOGY AND PATHOPHYSIOLOGY

MDD is thought to be caused by complex processes involving biological, psychological, and social factors (Dowrick, 2013; Garcia‐Toro & Aguirre, 2007; Kendler et al., 1993; Schotte et al., 2006). Existing observational research shows some evidence linking genetics (Ising & Holsboer, 2006), immunological (Segerstrom & Miller, 2004), hormonal, neurological, and neuroendocrinological body mechanisms to stress response, an important etiologic risk factor of MDD (Kendler et al., 1993; Wang, 2005). Evidence regarding the functional changes in the brain causally related with depressive symptoms is mixed (Hasler, 2010). It has been suggested that multiple factors, such as genetic vulnerability (Sullivan et al., 2000), altered hypothalamic–pituitary–adrenal (HPA) axis function (Dantzer et al., 2008), deficiency of monoamines (Kirsch et al., 2002; Speerforck et al., 2014), dysfunction of specific brain regions (Kerestes et al., 2014; Maletic et al., 2007), neurotoxic and neurotrophic processes (Serafini et al., 2014), altered glutamatergic and GABAergic neurotransmission (Hasler et al., 2007), dysregulation of glutamate system (Hasler et al., 2007; McEwen et al., 2012), and impaired circadian rhythms (Golden et al., 2005; Povitz et al., 2014), may have independent and cumulative effects that mediate or moderate each other's effects to cause MDD symptoms. Some cohort studies have suggested that age at first onset (which can occur at any time) may reflect different causal mechanisms (Burke et al., 1991; Kessler et al., 2007; Weissman et al., 1988). First‐time diagnosis during childhood may be indicative of genetic predisposition (Hazell, 2002a; Rice et al., 2002) or exposure to psychosocial childhood adversity (Hazell, 2002a). During adolescence, etiology has been mainly attributed to psychosocial and economic factors (Birmaher et al., 1996; Hazell, 2002b). At this age, a disparity in MDD incidence and prevalence by sex emerges with significantly higher incidence and prevalence among girls than boys (Hankin & Abramson, 2001; Nolen‐Hoeksema & Girgus, 1994). Reasons for this disparity include differences in biological body mechanisms, stress sensitivity, culture, and stress coping strategies between males and females (Nolen‐Hoeksema, 1991; Nolen‐Hoeksema et al., 1991; Shih et al., 2006). This gender disparity in morbidity persists into adulthood. Women as a function of changing biological and hormonal factors remain at high risk of MDD during their childbearing years (Kessler, 2003; Kessler et al., 1994) particularly during prenatal and postnatal periods.

## 3 EFFECTS OF MATERNAL MDD

Among pregnant women, MDD negatively affects fetus health (Chung et al., 2001; Dieter et al., 2008; Kinsella & Monk, 2009). For example, pregnant women with MDD have been shown to have a higher fetal heart rate (FHR) than women without MDD. Following vibratory stimulation tests (i.e., clinical assessments of fetal health), fetuses of pregnant women with MDD have been shown to have delayed FHR habituation while fetuses of pregnant women without MDD had startle fetus reflex and accelerated FHR or transient tachycardia (Allister et al., 2001; Sandman et al., 2003). Among pregnant women with MDD, the higher baseline FHR and delayed habituation poststimulation are associated with HPA dysregulation (linked to higher levels of glucocorticoid transfer from mother to fetus) that negatively impact fetal development (Gilles et al., 2018; Sandman et al., 2003). Higher levels of fetal glucocorticoid exposure are associated with lower birth weight and shorter gestation at delivery (Gilles et al., 2018). Similar findings supportive of a causal hypothesis were reported in a prospective cohort study examining the association between FHR and general psychosocial stress, a risk factor for maternal MDD (DiPietro et al., 1996). Overall, MDD during pregnancy is linked to increased risk of negative obstetric and neonatal outcomes such as preeclampsia, premature delivery, and low birth weight (Buss et al., 2012; Chung et al., 2001). During the postnatal period, these effects may be compounded by poor mother–child interactions and nurturing among mothers with MDD putting children at high risk of infant morbidity and mortality (as a function of either neglect or abuse), delay in meeting appropriate development milestones, and behavioral problems (Lovejoy, 1991; Surkan et al., 2012, 2014).

## 4 MATERNAL MDD DETECTION AND DIAGNOSIS

Similar to a diagnosis of MDD in the general population, maternal MDD is not an objective diagnosis because it is in part based on subjective experiences and perceptions. As a consequence of its subjective nature, a number of different maternal MDD tools have been adopted for screening, case‐finding, and diagnosis as well as for monitoring treatment progress (Myers et al, 2013). The operational definitions of MDD under these tools typically involve a count and weighting of symptoms that are present over a period of 1 or 2 weeks. The number of symptoms present (including their severity ratings) is used to set a threshold above which a patient meets the MDD operational definition (Gaynes et al., 2005; Myers et al., 2013; Pignone et al., 2002). Often, the diagnostic performance of these case‐finding tools is confounded by different perceptions, cultures, and assessment periods—prenatal versus postnatal (Horwitz et al., 2007; Owora et al., 2016b). Indeed, maternal MDD is often under‐ or overdiagnosed due to the presence of symptoms that mimic those of normal prenatal and postnatal periods (Owora et al., 2016b). Heterogeneity has also been demonstrated in existing diagnostic accuracy studies (Owora et al., 2016a, 2016b) in part due to clinical diversity (i.e., differences between study participants) and methodological diversity (i.e., differences in the measurement, timing, and definition of MDD). These differences have important implications for the validity of case‐finding tools used to classify mothers as either MDD‐positive or ‐negative. Some studies (Levis et al., 2020) have attempted to address potential misclassification by using higher cut‐off values and/or redesign of self‐reported questions (e.g., Edinburgh Postnatal Depression Scale) to reduce confusion between MDD and normal prenatal and postpartum symptoms, with mixed results for the reduction of false‐positives and ‐negatives.

## 5 MATERNAL MDD: INDIVIDUAL ‐ AND POPULATION‐LEVEL INFERENCE

In psychiatry, there continues to be a paucity of research on the impact of imperfect case‐finding tools on individual‐ and population‐level inference. Yet, if unaccounted for, misclassification of psychiatric disorders, such as MDD, can lead to inappropriate clinical decisions in patient care (e.g., treating or referring a patient without MDD for further diagnostic work‐up or failing to do so for patients with MDD). Such misclassification may be more prevalent among nonspecialist than specialist clinicians in primary care and/or public health prevention program settings (Horwitz et al., 2007; Myers et al, 2013).

At the population level, estimation of disease burden or risk is hampered with direct implications for allocation of public health resources and design or targeting of prevention efforts, respectively. The argument that the use of existing case‐finding tools results in misclassification bias is not new; in fact, it has been argued for decades, but their implications and particularly how to correct for these errors for valid inference is unexplored.

In this perspective piece, I revisit why MDD measurement errors or bias are important to consider from a clinical and public health view using recent epidemiologic studies. This article is not intended to be a comprehensive review; rather, I have deliberately selected articles, some of which are my own (Owora & Carabin, 2018; Owora et al., 2019, 2016b), to illustrate how existing estimates of maternal MDD prevalence, case‐finding tool sensitivity, and specificity can be used to generate accurate risk, burden, and measures of association to inform valid individual‐ and population‐level inference.

## 6 CORRECTING FOR MISCLASSIFICATION ERRORS TO MAKE INDIVIDUAL‐LEVEL INFERENCE

The concept of quantifying perceptions or impressions in clinical decision making, especially regarding diagnosis and prognosis, is not new. For instance, the likelihood of a specific diagnosis (i.e., the presence or absence of disease) is particularly appealing in the absence of confirmatory diagnostic testing. To evaluate a disease hypothesis based on nonconfirmatory test results, a positive predictive value (PPV) is defined as the probability of disease (e.g., MDD) given a positive test result (e.g., a *Center of Epidemiological Studies‐Depression 20‐item questionnaire [CESD20]* with a moderate or severe score ≥16). Conversely, a negative predictive value (NPV) is defined as the probability of no disease given a negative test result (e.g., no or few MDD‐related symptoms reported on the *CESD20*).

When combined with disease prevalence or pretest probability of disease (*P_D_*) and known test properties, such as sensitivity (*Se*) and specificity (*Sp*) using Bayes theorem, conditional probabilities (PPV and NPV) and likelihood ratios can be used to make individual‐level inference about the probability of disease given a test result, that is, P (D|T).

(1) $$\begin{array}{ccc} \mathrm{PPV} & = & \mathrm{True}\;\mathrm{Positives}\left[\mathrm{TP}\right]÷\left(\mathrm{TP}+\;\;\mathrm{False}\;\mathrm{Positives}\left[\mathrm{FP}\right]\right)\\ & = & P_{D}Se÷\left(P_{D}Se+\left[1-P_{D}\right]\cdot \left[1-Sp\right]\right), \end{array}$$

(2) $$\begin{array}{ccc} \mathrm{NPV} & = & \mathrm{True}\;\mathrm{Negatives}\left[\mathrm{TN}\right]÷\left(\mathrm{TN}+\;\mathrm{False}\;\mathrm{Negatives}\left[\mathrm{FN}\right]\right)\\ & = & \left(1-P_{D}\right)Sp÷\left(\left[1-P_{D}\right]Sp+\left[P_{D}\right]\cdot \left[1-Se\right]\right). \end{array}$$

Evidently, the higher the disease prevalence ($P_{D}$), the higher we expect PPV and NPV values. Moreover, the calculation of these values is expected to vary by test score cut points used to define MDD status.

On the other hand, likelihood ratios provide an intuitive and straightforward interpretation. The likelihood ratio is a ratio of two conditional probabilities—probability of a positive (or negative) test result given that the disease is present (or absent). Therein, two variants of the likelihood ratios are needed, one for if an individual's test is positive (positive likelihood ratio: LR^+^) and another if an individual's test is negative (negative likelihood ratio: LR‐).

(3) $${\mathrm{LR}}^{+}=P_{\mathrm{TP}}÷P_{\mathrm{FP}}=Se\;÷\left(1-Sp\right),$$

(4) $$\mathrm{LR}-=P_{\mathrm{FN}}÷P_{\mathrm{TN}}=\left(1-Se\right)\;÷Sp.$$

Applied to MDD, the post‐test probability of disease (i.e., P (D|T) can be derived from the post‐test odds (i.e., product of the pre‐test odds and likelihood ratio) as:

(5) Post-testodds=pre-testoddsxlikelihoodratio

and

(6) $$\mathrm{Post}-\mathrm{test}\;\mathrm{probability}=\mathrm{Post}-\mathrm{test}\;\mathrm{odds}/\left(1+\mathrm{post}-\mathrm{test}\;\mathrm{odds}\right),$$

where

(7) $$\begin{array}{ccc} \mathrm{odds} & = & \mathrm{probability}\;\mathrm{of}\;\mathrm{having}\;\mathrm{MDD}\left(P_{D}\right)/1\\ & & -\mathrm{probability}\;\mathrm{of}\;\mathrm{having}\;\mathrm{MDD}\left(P_{D}\right) \end{array}$$

and

(8) $$\text{The}\;\text{probability}\;\text{of}\;\text{having}\;\text{MDD}\left(P_{D}\right)=\mathrm{odds}/\left(1+\mathrm{odds}\right).$$

Assuming, *Se* is 80% and *Sp* is 90% and a prevalence of MDD in a hypothetical population is 20%, the LR+ = 8 (i.e., 0.8/[1–0.9] from Equation 3) and LR‐ = 0.2 (i.e. [1–0.8]/0.9 from Equation 4) and pretest odds = 0.25 (derived from 0.2/[1–0.2] from Equation 7), two scenarios may be developed to illustrate the use of the likelihood ratio.

*Scenario one*: If a woman tested positive for MDD based on a case‐finding tool at a defined score threshold, her post‐test odds of having MDD would be 0.25 × 8 = 2 (*substitution into Equation* **5**: pretest odds × LR^+^).

Substitution into Equation **6**: Post‐test probability of having MDD given a positive test (i.e.,$P\left(D^{+}|T^{+}\right)=2/\left[1+2\right]=0.67\left(67\%\right)$.

These results indicate that after testing positive for MDD, a woman's probability of having a MDD diagnosis is increased by 47% (i.e., from 20%—the population prevalence). While being mindful of other conditions that may mimic, cause, or coexist with MDD, a higher post‐test probability would warrant further testing to confirm or rule out a MDD diagnosis. However, recommendations and referral to psychotherapy and/or prescription of antidepressants as suggested by the American Psychiatric Association and American College of Obstetricians and Gynecologists is warranted (Myers et al., 2013; O'Hara & McCabe, 2013).

In addition to a positive MDD test result, if a woman was pregnant and aged 18–25 years old, her pretest probability would be higher than 20% (population prevalence) since national prevalence estimates show that pregnant women who are 18–25 years old have a MDD pretest probability of 36% (Zhou et al., 2019). Consequently, because such a woman's pretest probability of MDD is greater than 20%, her post‐test probability of having MDD given a positive test result will be 15% higher than that for a woman from the general population (i.e., 82% derived by the direct application of Equations **5**–**8**).

*Scenario two*: If a woman's case‐finding tool result is negative for MDD, her post‐test odds of having MDD would be 0.25 × 0.2 = 0.05 (*substitution into Equation* ***5***: pretest odds × LR^−^).

Substitution into Equation **6**: Post‐test probability of having MDD (i.e., $P\left(D^{+}|T^{-}\right)=0.05/\left[0.05+1\right]=0.05\left(5\%\right)$

Considering the American Psychiatric Association and American College of Obstetricians and Gynecologists guidelines and recommendations, without any other stressors or risk factors (e.g., history of MDD), such a woman may not necessarily warrant additional follow‐up. It should be noted, however, that the above example is only for illustration purposes and is not a substitute for a full clinical workup and differential diagnoses, but hopefully augments that process for better clinical judgment among mental healthcare providers.

It is important to note, however, that the use of the likelihood ratio approach for individual‐level inference is not without its own limitations. For example, (1) a given LR+ (e.g., 10) value can be generated from different combinations of S*e* and S*p* (e.g., 10 and 99% or 40 and 96%, respectively); (2) LRs are not linear (i.e., formula involves a division “÷” arithmetic operation); and (3) precision of high and low LRs is low. Despite these limitations, the translation of the likelihood ratio approach for individual‐level inference using a nomogram (Fagan, 1975) can enhance its clinical utility. If the prevalence of disease and likelihood ratios are known, one can easily find the P (D|T) associated with a particular test result (±).

## 7 CORRECTING FOR MISCLASSIFICATION ERRORS TO MAKE POPULATION LEVEL INFERENCE

### 7.1 Prevalence estimation

In our recent article, we illustrate the prevalence estimation problem using results of the *CESD20* (Owora & Carabin, 2018). Based on recent meta‐analysis results (Owora et al., 2016b), the *CESD20* is estimated to have on average, a sensitivity (S*e*) of 84% and specificity (S*p*) of 78% for identifying patients with moderate or severe MDD symptoms based on a total score cut point of 16. If the *CESD20* were administered to 1000 women in a population with a “true” MDD prevalence of 10%, we expect results shown in Table 1.

**TABLE 1:** Contingency table for the “true” and observed results derived from the CESD20

		True MDD Diagnosis		
		Positive	Negative	
*CESD20* RESULTS	Positive (≥16)	84 (TP)	198 (FP)	282 (T^+^)
	Negative (<16)	16 (FN)	702 (TN)	718 (T^−^)
		100 (D^+^)	900 (D^−^)	

P_TP_ = P(T^+^|D^+^) x P(D^+^) = S*e* x P(D^+^).

P_FP_ = P(T^+^|D^−^) x P(D^−^) = (1 ‐ S*p*) x (1− P(D^+^)).

P_FN_ = P(T^−^|D^+^) x P(D^+^) = (1‐S*e*) x P(D^+^).

P_TN_ = P(T^−^|D^−^) x P(D^−^) = S*p* x (1− P(D^+^)).

In this case, the estimated (biased) prevalence of MDD would be 28.2% (282/1000) which is 18.2% higher than the “true” prevalence (misclassification bias or error = 28.2−10%). The PPV of 29.8% (i.e., 84 of the 282 positive tests truly have MDD) warrants a cautious interpretation of *CESD2‐*positive test results to avoid the overestimation of MDD burden.

To correct for such misclassification error, if we assume T^+^ represents the number of individuals who test positive for MDD using the *CESD20* and D^+^ represents the number of individuals who truly have MDD then the conditional probability of an individual testing positive given that an individual truly has MDD is equal to the probability of truly having MDD and testing positive divided by the probability of truly having MDD denoted as:

(9) $$\begin{array}{ccc} P\left(T^{+}|D^{+}\right) & = & P\left(D+nT+\right)/P\left(D^{+}\right)P\left(T^{+}|D^{+}\right)\\ & = & P\left(D^{+}|T^{+}\right)P\left(T^{+}\right)/P\left(D^{+}\right). \end{array}$$

Using Bayes’ theorem, if we assume a gold standard test for MDD exists, we can describe the association between the observed and true status as follows:

(10) $$P\left(T^{+}\right)=P\left(T^{+}|D^{+}\right)P\left(D^{+}\right)+P\left(T^{+}|D^{-}\right)P\left(D^{-}\right),$$

where P(T^+^) corresponds to the proportion of individuals testing positive for MDD (observed prevalence), P (T^+^|D^+^) corresponds to the sensitivity of the test (*Se*), P (T^+^|D^−^) corresponds to one minus the specificity of the test (*1‐Sp*), and P (D^+^) to the true prevalence of MDD.

(11) $$\begin{array}{ccc} P\left(D^{+}\right) & = & \left(P\left(T^{+}|D^{+}\right)+P\left(T^{-}|D^{+}\right)\right)/\left(P\left(T^{+}|D^{+}\right)+P\left(T^{+}|D^{-}\right)\right.\\ & & \left.+\;P\left(T^{-}|D^{-}\right)+P\left(T^{-}|D^{+}\right)\right). \end{array}$$

In our previous study (Owora & Carabin, 2018), we extend these concepts in a Bayesian latent class model to demonstrate that ignoring the misclassification error of case‐finding tools (e.g., *CESD20*) when estimating MDD prevalence among pregnant and postpartum women can result in an underestimation of the true MDD prevalence with misclassification bias (i.e., difference between adjusted and observed prevalence estimates) ranging from 6 to 43%, depending on the distribution of pre‐ versus postnatal assessments. Such bias can lead to the misappropriation of scarce resources to tackle the issue of MDD among mothers.

### 7.2 Risk factor measures of association

As an extension to the above discussion, unbiased measures of association between MDD and suspected risk factors are critical to identifying modifiable factors upon which preventive interventions can be developed. In our companion article (Owora et al., 2019), we demonstrate that adjustment for misclassification error in risk association studies is possible with a direct extension of the estimation concepts covered above to incorporate comparison of misclassification error‐adjusted prevalence estimates between risk factor (E^+^ or E^−^) categories (from Equation **11** above: P (D^+^)_E+_/P (D^+^) _E‐_) to generate a prevalence proportion ratio. Specifically, we show that failure to adjust for case‐finding tools’ misclassification error can lead to the underestimation of the effects of some risk factors (e.g., intimate partner violence) or the overestimation of others (e.g., period of MDD assessment: pre‐ vs. postnatal) on maternal MDD with varying magnitudes depending on the overall demographic and clinical profile of an investigated study sample.

For illustration, if we assume a simple scenario with nondifferential misclassification of MDD among pregnant and nonpregnant women (i.e., S*e* and S*p* are the same for both pregnant and nonpregnant women at 84 and 93%, respectively). In a hypothetical study sample (Table 2) with 1000 women, where 46% are pregnant, and 10% get a positive CESD20 test result, the observed risk of MDD would be 88% higher among the pregnant than nonpregnant women (risk ratio: 1.8; 95%CI: 1.2, 2.6).

**TABLE 2:** Contingency table for the hypothetical observed CESD20 test results by pregnancy status

		Pregnant?		
		Yes	No	
***Observed CESD20***	Positive (≥16)	60 (a)	40 (b)	**100**
	Negative (<16)	400 (c)	500 (d)	**900**
		**460 (E^+^)**	**540 (E^−^)**	

Applying our knowledge of S*e* and S*p*, we can calculate adjusted estimates for each cell using the Equations 12 and 13 to generate adjusted results in Table 3, with an adjusted risk ratio of 14.1 (95% CI: 4.4–45.4)

(12) $$A=\left(a-E^{+}\left[1-Sp\right]\right)/\left(Se-\left[1-Sp\right]\right),$$

where *C* = *E* ^+^ − *A*

(13) $$B=\left(b-E^{-}\left[1-Sp\right]\right)/\left(Se-\left[1-Sp\right]\right),$$

where *C* = *E^−^* − *B*.

**TABLE 3:** Contingency table for the adjusted CESD20 test results by pregnancy status

		Pregnant?		
		Yes	No	
***Corrected CESD20***	Positive (≥16)	36 (A)	3 (B)	**39**
	Negative (<16)	424 (C)	537 (D)	**961**
		**460 (E^+^)**	**540 (E^−^)**	

In real life, bias can involve more the just the measurement of the outcome of interest but also exposures, confounders, mediators, or moderators; these misclassification errors can be with either nondifferential or differential (i.e., S*e* and S*p* are different for E^+^ and E^−^). Moreover, these variables can be either categorical or continuous. The correction of misclassification bias (or measurement error) can involve a simple bias correction (Lash et al., 2009) to more complex approaches that include probabilistic bias correction (Fox et al., 2005), Bayesian bias‐correction (MacLehose et al., 2009), modified maximum likelihood (Edwards et al., 2014), and multiple imputation (Cole et al., 2006), propensity score (Lunt et al., 2012), and/or regression calibration (Rosner et al., 1989).

In summary, interest in the validity of MDD case‐finding tools among mothers of young children during the pre‐ and postnatal periods is well justified (Owora & Carabin, 2018; Owora et al., 2019, 2016a, b). There is a growing recognition of the multiple cross‐cutting negative effects of MDD on maternal–child health during the critical developmental stages of a child (Ammerman et al., 2010; Chung et al., 2004; Heckman, 2006; Lyons‐Ruth et al., 1990; Sills et al., 2007; Stewart & Vigod, 2019; Thombs et al., 2014; Whitaker et al., 2006). Errors in detection (i.e., false‐positive and ‐negatives) can result in initiation of unnecessary treatment or failure to treat maternal MDD. Valid population‐level inference related to incidence or prevalence and risk factor measures of association are critical to informing appropriate allocation of scarce healthcare resources and identifying modifiable factors for preventive intervention, respectively. Given the availability of methods that can be used to correct for MDD misclassification errors or bias derived from imperfect case‐finding tools, we recommend that the correction for these errors in clinical, public health practice, and research should be the default option, and not the exception.

## CONFLICT OF INTERESTS

Arthur H. Owora (author) has no conflict of interest to declare.

### PEER REVIEW

The peer review history for this article is available at https://publons.com/publon/10.1002/brb3.2614

## Data Availability

The data that support the findings of this study are available from the corresponding author upon reasonable request.
